# Variation in emergency department attendances and acute hospital admissions for ambulatory emergency care: a retrospective analysis of routinely collected NHS data across England

**DOI:** 10.1136/bmjopen-2025-115446

**Published:** 2026-06-23

**Authors:** Richard M Jacques, Rebecca M Simpson, Madina Hasan, Ric Campbell, Simone Croft, Susan Croft, Sophie Williams, Suzy Gallier, Felicity Evison, Amy Dillon, Ben Glampson, Quinta Davies, Jo Knight, Cai Davis, Michael George, Charles Gutteridge, Elizabeth Sapey, Rachel Denholm, Erik Mayer, Vishnu Chandrabalan, Matt Stammers, Suzanne Mason, HDRUK UK Regional Linked Data Consortium

**Affiliations:** 1 School of Medicine and Population Health, The University of Sheffield, Sheffield, UK; 2 Data Connect, The University of Sheffield, Sheffield, UK; 3 Sheffield Teaching Hospitals NHS Foundation Trust, Sheffield, UK; 4 Barts Life Sciences, Barts Health NHS Trust, London, UK; 5 PIONEER Data Hub in Acute Care, University Hospitals Birmingham NHS Foundation Trust, Birmingham, UK; 6 NIHR Midlands Patient Safety Research Collaboration and NIHR Biomedical Research Centre, University Hospitals Birmingham NHS Foundation Trust, Birmingham, UK; 7 Population Health Sciences, Bristol Medical School, University of Bristol, Bristol, UK; 8 NIHR Bristol Biomedical Research Centre, University of Bristol, Bristol, UK; 9 Faculty of Medicine, Department of Surgery and Cancer, Imperial College London, London, UK; 10 Imperial Clinical Analytics Research & Evaluation (iCARE) Secure Data Environment, NIHR, Imperial BRC, Imperial College Healthcare NHS Trust, London, UK; 11 Department of Data Science, Lancashire Teaching Hospitals NHS Foundation Trust, Preston, UK; 12 Lancaster Medical School, Lancaster University, Lancaster, UK; 13 Southampton Emerging Therapies and Technologies (SETT) Centre, University Hospital Southampton NHS Foundation Trust, Southampton, UK; 14 Clinical Informatics Research Unit (CIRU), University of Southampton, Southampton, UK; 15 Clinical Informatics, Barts Health NHS Trust, London, UK; 16 Health Data Research UK South-West, Bristol, Bristol, UK; 17 HDR UK, London, UK

**Keywords:** Emergency Departments, Observational Study, ACCIDENT & EMERGENCY MEDICINE

## Abstract

**Abstract:**

**Objectives:**

Rising demand for emergency care in England is a continuing challenge driven by population ageing and increasing multimorbidity. Ambulatory emergency care (AEC) refers to the provision of same-day acute care for patients who might otherwise require admission. However, the contribution of AEC conditions to demand remains unclear. This study aimed to examine the proportion and nature of patients attending emergency departments (ED) with AEC-related conditions and to describe variation between hospitals in attendances and emergency admissions for AEC conditions.

**Design and setting:**

A retrospective study of routine data from 21 acute hospitals in England, including adult ED attendances and emergency admissions between 1 November 2021 and 31 October 2022. We used a federated approach to ensure data security, applying established AEC definitions to explore variation by age, socioeconomic status and length of stay.

**Outcome measures:**

Primary: Proportion of (i) ED attendances and (ii) emergency admissions for AEC conditions. Secondary: (i) Proportion of patients presenting at ED with an AEC condition who were admitted; (ii) proportion of emergency admissions with an AEC condition with a length of stay <2 days.

**Results:**

We analysed 1 513 480 attendances (median per hospital: 73 125) and 660 105 admissions (median per hospital: 30 425). AEC accounted for 29.6% of attendances and 40.8% of admissions, with substantial inter-hospital variability. Patients aged ≥65 were more likely to present with an AEC, while patients from deprived areas had lower rates. Among AEC-related admissions, 49.3% had a stay of less than 2 days.

**Conclusions:**

Nearly one-third of attendances and two-fifths of admissions were for conditions potentially manageable in AEC or community settings. Variation between hospitals suggests local factors, including service configuration and primary care access, may influence avoidable acute care use. These findings suggest a need for a more nuanced understanding of the drivers behind AEC, or SDEC Services, to better understand their impact on reducing hospital admissions. Analysing these patterns may inform interventions to reduce avoidable hospital utilisation. Further research is needed to identify drivers of variation and to develop scalable strategies for prevention.

**STRENGTHS AND LIMITATIONS OF THIS STUDY**

- A large multi-centre observational study of more than 1.5 million emergency department attendances and 660 000 emergency admissions from 21 hospitals across England.
- A ‘federated’ approach where patient-level datasets remained within seven regional secure data environments enabled multi-site analysis while complying with UK information-governance requirements.
- The federated approach required methodological simplicity and limited the ability to perform case-mix adjustment. Therefore, some of the observed variability may be due to differences in population age, frailty, morbidity and social deprivation rather than service configuration.
- The federated approach meant that post hoc sensitivity analysis or further investigation of underlying causes could not be performed.

## Introduction

 Rising demand for emergency care has been widely documented within the English NHS. National statistics show that total emergency department (ED) attendances have grown substantially over the past decade from 22.4 million in 2014–15 to 27.4 million in 2024–25.^1^ Importantly, recent NHS England data demonstrates that increases in attendances are observed even after accounting for population growth, with ED attendances rising from 28 014 per 100 000 population in 2022–23 to 28 706 per 100 000 population in 2024–25.^1^,^3^ This trend reflects structural pressures, including population ageing and increasing clinical complexity, evidenced by rising attendance rates among older age groups and those with multimorbidity,^4^ alongside significant constraints in access to community and primary care services.^5^

Considerable uncertainty remains regarding the proportion of ED attendances and emergency admissions attributable to conditions potentially manageable through ambulatory or same-day emergency care (SDEC) pathways. While previous research has focused on ambulatory care sensitive conditions (ACSC), there has been less focus on ambulatory emergency care (AEC), which refers to the provision of same-day acute clinical care, including assessment, investigation, treatment and rehabilitation, for patients who might otherwise require inpatient admission.^6^,^8^

ACSC and AEC conditions represent heterogeneous clinical groups, and admission decisions are strongly influenced by local service configuration, diagnostic capacity, workforce availability and risk tolerance rather than diagnosis alone.^9 10^ Evidence from England demonstrates substantial and persistent geographical variation in emergency admissions for ACSCs, with both between-system and within-system differences remaining after adjustment for age and sex, suggesting that organisational provider-level factors play an important role.^11^

Variation is also observed at the hospital level, where outcomes and patterns of emergency admissions for similar conditions differ significantly between providers, implying that local clinical pathways and operational practices influence whether patients are admitted, managed through AEC or discharged.^12^ Policy analyses similarly note that many admissions classified as potentially avoidable or urgent-care sensitive are clinically necessary in some contexts and that stable population-adjusted admission rates despite rising overall emergency activity indicate uncertainty regarding the true scope for substitution away from inpatient care.^13^ Together, these findings suggest that estimates of avoidable admissions should be interpreted cautiously, as the boundary between ED management, AEC and inpatient admission varies across hospitals and health systems.

This study examines variation between hospitals in England in ED attendances and emergency hospital admissions for AEC conditions. In doing so, it explores methods for identifying clinically preventable emergency care and highlights opportunities to reduce avoidable acute service use.

## Methods

### Study design

A retrospective, observational study of ED attendances and emergency admissions at 21 acute hospitals in England between 1 November 2021 and 31 October 2022 using routinely collected data.

### Data collection

Using a ‘federated’ approach, ED attendance and hospital admission data were extracted, processed and analysed at seven regional centres covering 21 acute hospitals (table 1). The regional centres covered a wide geography, incorporating urban, semi-urban and rural locations. ‘Federated’ is used here to describe an approach whereby patient-level datasets remain within each regional centre’s data environment, with only summary statistical output shared for synthesis. This enabled multi-site analysis while complying with UK information-governance requirements that restrict central pooling of identifiable health data.

**Table 1:** Description of underlying populations at each centre

*Analysis centre*	*Region*	*Integrated care board*	*Population size* (*millions*)	*Hospital trusts*	*Hospitals*
Barts Health	*London*	*NHS Northeast London*	**2.2**	**1**	**3**
Bristol	*South* *West*	*NHS Bristol, North Somerset and South Gloucestershire*	**1.0**	**2**	**2**
iCARE SDE	*London*	*NHS Northwest London*	**2.1**	**4**	**7**
Lancashire	*North* *West*	*NHS Lancashire and South Cumbria*	**1.8**	**1**	**1**
PIONEER	*West* *Midland*	*NHS Birmingham and Solihull*	**1.3**	**1**	**3**
Sheffield	*Yorkshire and the Humber*	*NHS South Yorkshire*	**1.4**	**4**	**4**
Southampton	*South* *East*	*NHS Hampshire and Isle of Wight*	**1.4**	**1**	**1**

To ensure consistent data extraction and processing across regions, data specification and processing documents were created by the lead research centre (Sheffield). The data specification was aligned with the nationally mandated Commissioning Data Set 6.2 Type 011: Emergency Care^14^ and the nationally mandated Commissioning Data Set 6.2 Type 130 Admitted Patient Care—Finished General Episodes.^15^ The data-processing document described how codes from the Emergency Care and Admitted Patient Care datasets should be categorised and was based on the corresponding NHS Digital Technical Output Specifications^16 17^

### Ethics

The study was classified as a service evaluation with individual patient-level data accessed and linked with approval of local research ethics committees and Caldicott guardians. The study used routine electronic healthcare data sources that linked, de-identified and aggregated individual patient-level data that are held in participating regional Secure Data Environments and accessed by local study teams under existing local governance processes. Tables comprising aggregate data from each site underwent disclosure review and minimisation to reduce the risk of disclosure by the local information governance team in accordance with the Working Group for Safe Data Access Professionals Handbook on Statistical Disclosure Control for Outputs, with meta-analysis conducted by the lead site. In South Yorkshire, ethical approvals were granted by the University of Sheffield (reference number 050058). In Birmingham (PIONEER), ethical approvals were provided by the East Derby Research Ethics Committee (reference 20/EM/0158) and the Health Research Authority (reference number 279353). The Bristol Biomedical Research Centre sought advice and guidance from the Health Research Authority and Caldicott Guardians, utilising anonymous patient information collated by the Trust’s direct care team members for service evaluation purposes. At Imperial, access to patient-level data within the ICHT iCARE SDE was approved by the NIHR Imperial BRC data access and prioritisation committee (covered by database ethics—South West—Central Bristol Research Ethics Committee reference 21/SW/0120; IRAS project ID 282093). In Lancashire, access to data for this project was approved as a service evaluation by Lancashire Teaching Hospital NHS Foundation Trust. For Southampton, the Wessex Secure Data Environment holds section 251 ethics.

### Inclusion and exclusion criteria

ED attendance data were included for all adults (aged 18 years or older on the day of their attendance) with a first unplanned emergency care attendance for a new clinical condition or deterioration of a chronic condition at a major ED (defined as a consultant-led 24-hour service with full resuscitation facilities and designated accommodation for the reception of emergency care patients) within the study period. ED attendance data for patients that ‘did not wait’ were included, but this subgroup of patients was not specifically investigated as part of the study. ED attendance data were excluded if they were for a planned follow-up or an unplanned follow-up for the same or a related clinical condition within 7 days of the first emergency care attendance.

Patient data for completed emergency admissions for adults with an admission date within the study period were included. All methods of emergency admission were included in the data (ie, admissions from EDs, general practitioners and other sources).

### Identifying ambulatory emergency care conditions

The AEC Directory^18^ was used to identify International Classification of Disease (ICD10) codes relating to 24 general medical conditions that could be managed in an ambulatory manner. General medical conditions were chosen rather than other categories such as trauma and orthopaedics, general surgery, urology and obstetrics and gynaecology because these presentations are most commonly managed through AEC, clinical decision units or SDEC pathways, which are designed to assess and treat acute medical patients without overnight admission where clinically appropriate.^6 8^ The ICD10 codes were then mapped to SNOMED CT codes using the Emergency Care Data Set Enhanced Technical Output Specification produced by NHS Digital.^16^ In addition to SNOMED CT codes identified for AEC, the code for ‘condition unspecified’ was included because this is also a potential indicator for avoidable admission. See online supplemental material A for details of ICD10 and SNOMED CT codes used.

ED attendances for AEC conditions were identified using the SNOMED CT codes recorded in the primary diagnosis field. Emergency inpatient hospital admissions for AEC conditions were identified using the ICD10 codes recorded in the primary diagnosis field for the first episode in inpatient spells with an emergency method of admission.

### Outcomes

The primary outcomes of interest were:

1. The proportion of ED attendances that were for an AEC condition.
2. The proportion of emergency hospital admissions (from any source) that were for an AEC condition.

The secondary outcomes were:

1. The proportion of patients presenting at ED with an AEC condition who were admitted.
2. The proportion of emergency hospital admissions (from any source) with an AEC condition that were less than 2 days in length.

### Analysis

For this exploratory investigation, all analyses were descriptive and conducted at the hospital level, which served as the primary unit of analysis. Using a federated approach to analysis, each regional centre produced tables of aggregated summary statistics at a hospital level based on an analysis plan developed at the University of Sheffield. The primary outcomes were calculated as proportions using total ED attendances or total emergency admissions at each site as the denominators. Before transferring the tables of summary statistics to the University of Sheffield for aggregated analysis, statistical disclosure control rules were applied to suppress small numbers and ensure that the confidentiality of individuals was maintained. The rules were as follows: (1) for cells with n=0, report 0; (2) for cells with 0<N<10, report as <10; (3) exclude any values <10 from the calculation of percentages; (4) after suppression of small numbers, round all other numbers to the nearest 5.

Researchers at the lead site then aggregated these data to describe variation between hospitals and present the national picture. The primary and secondary outcomes were visualised using forest plots with the overall pooled proportions calculated, weighting according to the number of attendances or admissions at each hospital. Notably, no-population-based adjustments were performed. Therefore, variation was assessed in relation to hospital activity volumes rather than local population size. Further analysis investigated variation in outcomes at a national level by patient demographics (age, sex, quintile of Townsend Deprivation Index,^19 20^ and ethnicity) with the number and proportion of attendances and admissions reported for each group.

### Patient and public involvement

There was no patient or public involvement in this study.

## Results

The study population included data from 21 hospital sites, with 1 513 480 first-time ED attendances (hospital site median: 73 125 attendances; range: 32 880 to 1 14 190) and 660 105 acute admissions (hospital site median: 30 425 admissions; range: 10 625 to 51 905).

### ED attendances

**Figure 1:**
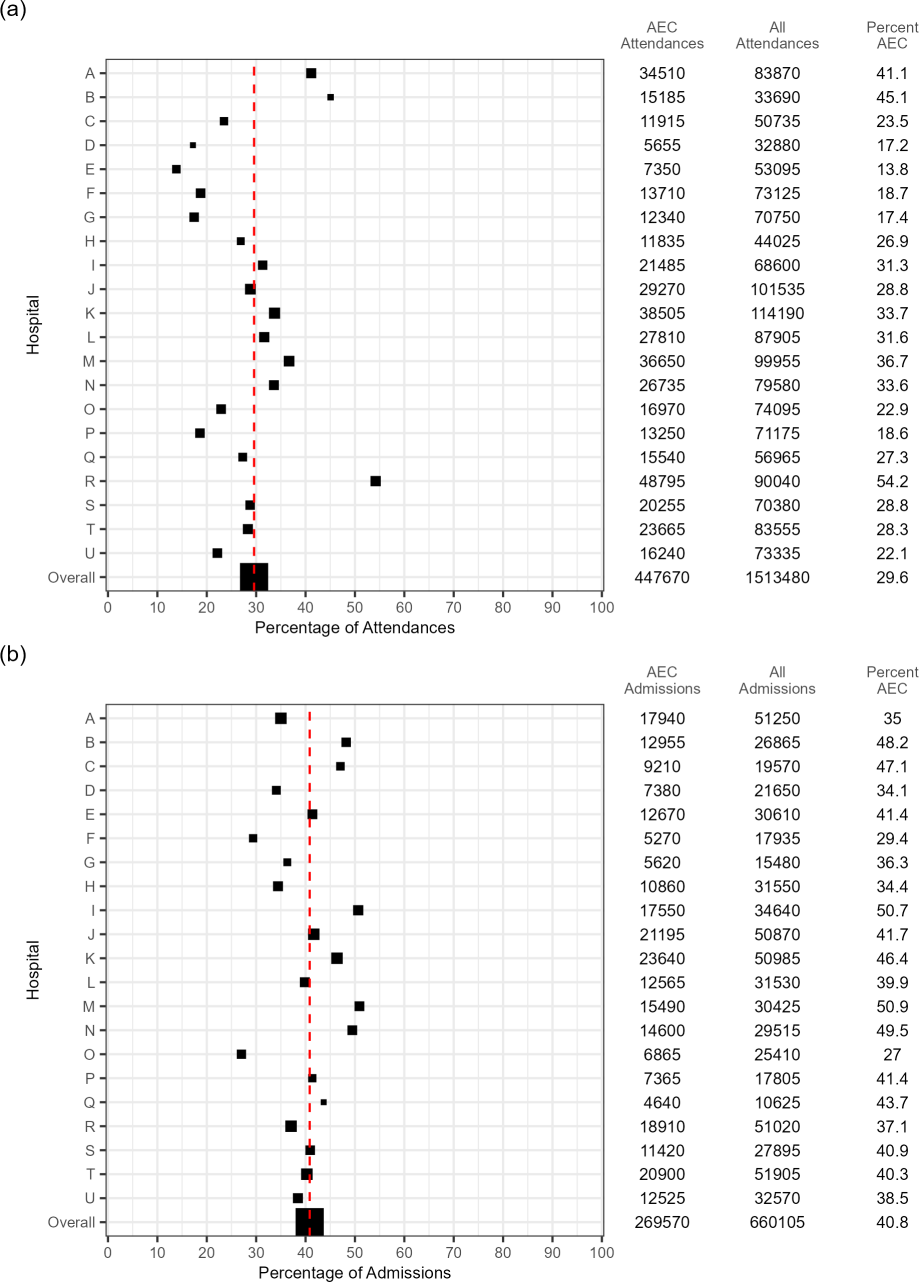
(**a**) Emergency department attendances and (**b**) acute hospital admissions for AEC conditions by hospital. AEC, ambulatory emergency care.

The proportion of ED attendances for AEC conditions is presented by hospital in figure 1a. Overall, 29.6% (447 670/1 513 480) of first-time ED attendances were for AEC. We found variation in the percentage of attendances for AEC by hospital, ranging from 13.8% (7 350/53 095) to 54.2% (48 795/90 040). Data on the type of AEC condition were available from 18 hospitals with 354 845 attendances for AEC. The most common diagnoses were condition unspecified (88 535; 25%) (where ‘condition unspecified’ would include conditions that were 'ruled out’ by the ambulatory care service (eg, DVT, PE ruled out—no diagnosis identified), lower respiratory tract infections/community-acquired pneumonia (31 965; 9%) and low-risk chest pain (31 770; 9%).

Table 2 shows the proportion of ED attendances for AEC by patient demographics. Attendances for patients aged 65 and over were more likely to be for AEC compared with the younger age groups. Specifically, 36.6% (120 170/328 175) of 65–84 year olds and 36.6% (41 820/114 295) of patients aged 85 or over had attendance for AEC, compared with 24.2% (163 945/676 640) of 18–44 year olds. The proportion of attendances for AEC was similar for females (30.8%; 242 755/787 575) compared with males (28.2%; 204 860/725 620). There was some variation in attendance for an AEC by deprivation, with the lowest proportion in the most deprived quintile (27.9%; 171 895/616 855) compared with the largest proportion in the least deprived quintile (33.7%; 42 270/125 400); and by ethnic group, with the lowest proportion in the other category (23.1%; 23 705/102 815) compared with the highest proportion in the white category (31%; 280 040/903 985).

**Table 2:** Emergency department attendances for ambulatory emergency care conditions by patient characteristics

	ED attendances		
	**All** **attendances,** **N**	**AEC attendances,** **N (%**)	**AEC** **admitted,** **N (%**)
**Age (years**)			
18–44	676 640	163 945 (24.2)	28 565 (17.4)
45–64	394 335	121 735 (30.9)	35 715 (29.3)
65–84	328 175	120 170 (36.6)	57 330 (47.7)
85+	114 295	41 820 (36.6)	24 680 (59.0)
Missing	35	0 (0.0)	0 (0.0)
Total	1 513 480	447 670 (29.6)	146 290 (32.7)
**Gender**			
Female	787 575	242 755 (30.8)	76 300 (31.4)
Male	725 620	204 860 (28.2)	69 990 (34.2)
Indeterminate/not known	205	25 (12.2)	0 (0.0)
Total	1 513 400	447 640 (29.6)	146 290 (32.7)
**Quintile of Townsend** **Deprivation Index**			
1 (least deprived)	125 400	42 270 (33.7)	16 435 (38.9)
2	153 465	50 940 (33.2)	18 930 (37.2)
3	207 645	66 615 (32.1)	24 010 (36.0)
4	305 900	94 160 (30.8)	30 995 (32.9)
5 (most deprived)	616 855	171 895 (27.9)	51 255 (29.8)
Missing	95 855	19 695 (20.5)	3895 (19.8)
Total	1 505 120	445 575 (29.6)	145 520 (32.7)
**Ethnicity**			
White	903 985	280 040 (31.0)	102 035 (36.4)
Asian	198 290	59 110 (29.8)	15 435 (26.1)
Black	88 920	23 660 (26.6)	6180 (26.1)
Mixed	24 420	5960 (24.4)	1395 (23.4)
Other	102 815	23 705 (23.1)	4630 (19.5)
Missing	195 115	55 215 (28.3)	16 610 (30.1)
Total	1 513 545	447 690 (29.6)	146 285 (32.7)

AEC, ambulatory emergency care; ED, emergency department.

**Figure 2:**
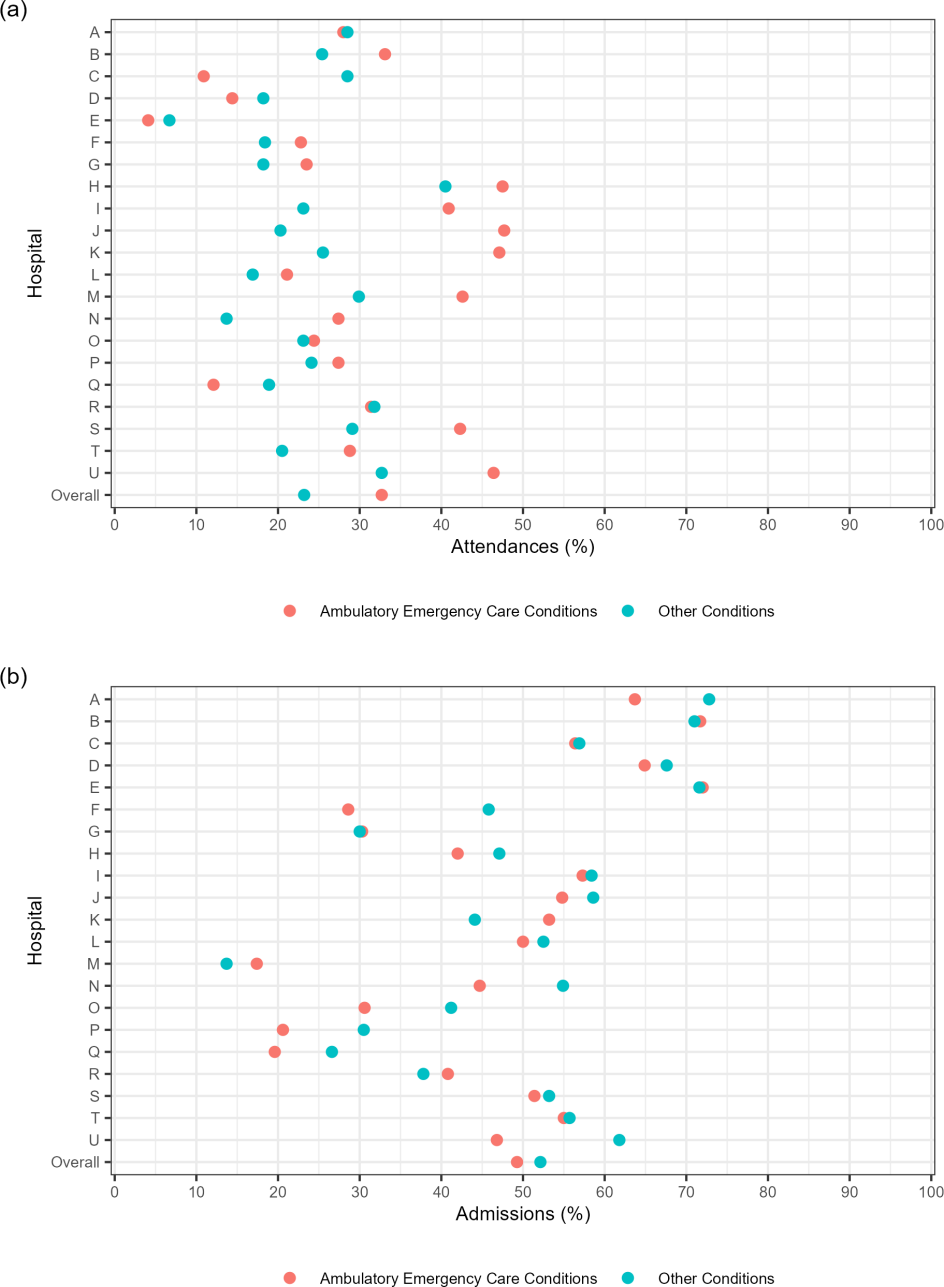
(a) Percentage of emergency department attendances that are admitted to hospital and (b) percentage of acute hospital admissions with length of stay less than 2 days.

Figure 2a shows the proportion of ED attendances for AEC conditions that result in hospital admission compared with attendances for other conditions. Overall, a higher proportion of attendances for AEC (32.7%; 146 315/447 645) result in admission compared with other conditions (23.2%; 247 055/1 065 255). We also found variation between hospitals in the proportion of admissions. The proportion of attendances for AEC resulting in admissions ranged from 4.1% (300/7350) to 47.7% (13 970/29 260), while attendance for other conditions resulting in admission ranged from 6.7% (3065/45 735) to 40.5% (13 050/32 200).

The proportion of ED attendances for AEC that result in hospital admission is presented by patient demographics in table 2. We found that the proportion of attendances resulting in hospital admission increased with age, with 17.4% (28 565/163 945) of 18–44-year-olds admitted compared with 59% (24 680/41 820) of patients aged 85 or over. The proportion of attendances resulting in admission was similar for females (31.4%; 76 300/242 755) and males (34.2%; 69 990/204 860) with some variability between index of multiple deprivation (38.9%; 16 435/42 270 of the least deprived admitted compared with 29.8%; 51 255/171 895 of the most deprived) and ethnicity (36.4%; 102 035/280 040 of white patients admitted compared with 19.5% 4630/23 705 of patients from other racial groups).

### Acute admissions

Figure 1b shows the proportion of acute admissions for AEC by hospital. Overall, 40.8% (269 570/660 105) of acute admissions were for AEC. We found variation in the percentage of admissions for AEC by hospital, ranging from 27% (6865/25 410) to 50.9% (15 490/30 425). Data on the type of AEC were available from 19 hospitals with 237 195 admissions for AEC. The most common diagnoses were low-risk chest pain (31 185; 15.2%), lower respiratory tract infections/community-acquired pneumonia (25,300; 10.7%) and falls, including syncope and collapse (18,745; 6.1%).

The proportion of acute admissions for AEC by patient demographics is shown in table 3. The proportion of admissions for AEC increased with age, with 29.7% (56 760/191 415) of 18–44-year-olds admitted with an AEC compared with 47.4% (39 815/84 085) of patients aged 85 and over. There was little variability in the proportion of admissions by deprivation, with the lowest proportion in the least deprived quintile (40.3%; 29 455/73 075) compared with the largest proportion in the third quintile (42.5%; 42 710/100 550). However, there was more variability in the proportion of admissions for AEC by ethnicity. The lowest proportion was observed in the other ethnic group (34.7%; 10 355/29 800) and the highest in the white ethnic group (42.3%; 176 005/416 415). The proportion of admissions for AEC was similar for females (40.5%; 141 970/350 815) and males (41.2%; 127 525/309 180).

**Table 3:** Acute hospital admissions for ambulatory emergency care conditions by patient characteristics

	Emergency admissions	
	**All** **admissions,** **N**	**AEC** **admissions,** **N (%**)
Age (years)		
18–44	191 415	56 760 (29.7)
45–64	168 200	71 030 (42.2)
65–84	216 405	101 965 (47.1)
85+	84 085	39 815 (47.4)
Not known	0	0 (0.0)
Total	660 105	269 570 (40.8)
Gender		
Female	350 815	141 970 (40.5)
Male	309 180	127 525 (41.2)
Indeterminate/not known	30	0 (0.0)
Total	660 025	269 495 (40.8)
Quintile of Townsend Deprivation Index		
1 (least deprived)	73 075	29 455 (40.3)
2	80 605	33 640 (41.7)
3	100 550	42 710 (42.5)
4	140 865	58 755 (41.7)
5 (most deprived)	226 435	92 180 (40.7)
Missing	33 550	11 085 (33.0)
Total	655 080	267 825 (40.9)
Ethnicity		
White	416 415	176 005 (42.3)
Asian	78 685	32 615 (41.5)
Black	31 525	11 745 (37.3)
Mixed	7725	2685 (34.8)
Other	29 800	10 355 (34.7)
Missing	95 940	36 115 (37.6)
Total	660 090	269 520 (40.8)

AEC, ambulatory emergency care.

Figure 2b shows the proportion of acute admissions for AEC with length of stay less than 2 days compared with admissions for other conditions. Overall, 49.3% (132 790/269 510) of admissions for AEC had a length of stay of less than 2 days compared with 52.1% (203 580/390 510) of admissions for other conditions. We also found variation between hospitals, with the proportion of admissions for AEC with a length of stay less than 2 days ranging from 17.4% (2,690/15,490) to 72% (9125/12 670) and admissions for other conditions ranging from 13.7% (2040/14 945) to 72.8% (24 250/33 310). We did not find evidence of a correlation between the proportion of ED attendances for AEC conditions that result in hospital admission and the proportion of acute admissions for AEC with length of stay less than 2 days (Spearman rho: −0.05, 95% CI −0.47 to 0.39).

## Discussion

In this multi-centre descriptive study of more than 1.5 million ED attendances and 660 000 emergency admissions across English hospitals, we found that conditions listed in the AEC Directory accounted for a substantial proportion of acute care activity. We found that 29.6% of first-time attendances at ED and 40.8% of acute admissions were included in the list of AEC-related conditions.

AEC conditions represent a heterogeneous group of presentations, many of which may appropriately require inpatient admission depending on patient complexity, diagnostic uncertainty or local service configuration. The findings therefore describe the potential population for ambulatory pathways rather than the proportion of admissions that could safely have been avoided.

A notable finding was that ED attendances coded as AEC conditions were more likely to result in hospital admission than attendances for other conditions. This pattern appears paradoxical given that AEC pathways are designed to support same-day management and avoid admission where clinically appropriate. One interpretation is that these diagnostic categories capture presentations that frequently require further investigation or short-term monitoring such as chest pain, suspected infection or syncope, leading clinicians to admit patients when ambulatory capacity options are limited. Alternatively, coding practices may capture provisional diagnoses recorded early in the patient pathway before diagnostic uncertainty is resolved. As a result, the higher admission rate observed for AEC conditions does not necessarily imply missed opportunities for ambulatory management but may instead reflect the complexity of clinical decision-making in emergency care.

The study also identified demographic differences in attendance and admission patterns. Older patients were more likely to present with and be admitted for AEC conditions, consistent with increasing multimorbidity and diagnostic complexity in ageing populations. Differences by deprivation and ethnicity were smaller but still present, suggesting potential challenges in healthcare access, health status or patterns of service use. These observations should be interpreted cautiously because the analyses were descriptive and did not adjust for clinical severity or comorbidity.

Our findings are consistent with previous research demonstrating substantial variation in emergency admissions for ACSCs across England. Analyses using national Hospital Episode Statistics have shown that admission rates for ACSCs vary markedly between local health systems even after adjustment for demographic characteristics, suggesting that organisational and system-level factors influence admission thresholds and care pathways.^9 21 22^

Internationally, studies have identified large regional differences in hospitalisations for ACSCs and proposed that these admissions may reflect differences in access to primary care and local practice patterns, particularly among older populations and individuals with multiple chronic conditions.^23^,^26^

Audits of AEC models in England have suggested that dedicated ambulatory services can support same-day discharge for a large proportion of patients assessed through SDEC pathways.^8^ However, the evidence base regarding the impact of SDEC services on admission rates remains heterogeneous. A recent systematic review of SDEC services identified predominantly single-centre observational evaluations and concluded that although these services may increase same-day discharge and improve patient flow, the evidence for sustained reductions in hospital admissions is limited and subject to significant risk of bias and confounding.^27^

The present study does not evaluate the effectiveness of AEC or SDEC services. Instead, it quantifies the proportion of ED attendances and emergency admissions associated with diagnostic categories included in the national AEC Directory. The findings therefore describe the potential population for ambulatory pathways rather than demonstrating that these admissions could have been safely avoided.

### Implications for policy and practice

The findings suggest several implications for policymakers. First, the scale of activity associated with AEC conditions indicates that these presentations represent an important target population for examining how emergency care pathways operate in practice. However, the results also highlight the limitations of relying on diagnostic categories alone to identify avoidable admissions.

The observed inter-site variation suggests that local system factors—such as the availability and effectiveness of community-based services, primary care access and care coordination may influence decisions to admit. While we do not posit that all admissions for AEC are avoidable, it is possible that a meaningful proportion could represent missed opportunities for prevention or community-based intervention, subject to further evaluation of clinical severity.

These findings support the case for further examination of alternative care pathways, including SDEC), ambulatory care units, virtual wards and enhanced pre-hospital triage. Identifying high-performing systems with lower avoidable admission rates may yield valuable insights into service models and resource configurations that mitigate unnecessary admissions. Strategic investment in out-of-hospital care and care navigation may, therefore, have the potential to contribute to system efficiency and patient-centred outcomes.

### Limitations

This study has several important limitations: (1) the federated analytical approach, while enhancing data privacy and site participation, required methodological simplicity and limited the ability to perform case-mix adjustment. Therefore, some of the observed variability between hospitals may be due to differences in population age, frailty, morbidity, and social deprivation rather than service configuration. The federated approach also meant that post hoc sensitivity analysis (such as excluding ‘condition unspecified’) or further investigation of underlying causes could not be performed. (2) There was variability in data quality and availability across hospitals. Despite extensive validation efforts, resource constraints limited the extent of standardisation and error checking. (3) The study used routinely collected retrospective data, and therefore, diagnostic uncertainty could not be investigated, although this might be implied through the high number of attendances that were coded as ‘condition unspecified’. (4) The diagnosis codes used to identify AEC may overestimate the avoidability of attendances and admissions, as they do not account for clinical nuance or patient preferences. More granular electronic health record data would help to understand the individual patient-level differences, such as observations, triage category and NEWS scores, driving decisions. (5) The sample only includes 21 hospitals, and although it covers different regions, it may not be representative of the entire English NHS system.

## Conclusions

This study reveals that a substantial proportion of ED attendances and acute hospital admissions are for conditions potentially amenable to SDEC or community-based management, with considerable variation observed between hospitals. These findings suggest a need for a more nuanced understanding of the drivers behind AEC or SDEC Services to better understand their impact on reducing hospital admissions. Future work should explore the variation observed between hospitals through examining the impact of local healthcare infrastructure, patient-level risk factors, clinical decision making and service availability. Ultimately, targeted interventions to reduce unnecessary admissions may benefit from a coordinated approach across primary, community and emergency care sectors.

## Supplementary material

10.1136/bmjopen-2025-115446 online supplemental file 1

## Data Availability

No data are available.
