# Supplementary material for: Variation in emergency department attendances and acute hospital admissions for ambulatory emergency care: a retrospective analysis of routinely collected NHS data across England
Source: BMJ Open. 2026 Jun 23;16(6):e115446. doi: 10.1136/bmjopen-2025-115446 (PMC13296041; doi:10.1136/bmjopen-2025-115446)
Supplement: Supplementary file 2 [file bmjopen-2025-115446.reviewer_comments.pdf]

PEER REVIEW HISTORY

BMJ Open publishes all reviews undertaken for accepted manuscripts. Reviewers are asked to complete a checklist review form and are provided with free text boxes to elaborate on their assessment. These free text comments are reproduced below.

ARTICLE DETAILS

Title (Provisional)

Variation in Emergency Department Attendances and Acute Hospital Admissions for Ambulatory Emergency Care: A Retrospective Analysis of Routinely Collected NHS Data across England

Authors

Jacques, Richard M; Simpson, Rebecca M; Hasan, Madina; Campbell, Richard; Croft, Simone; Croft, Susan; Williams, Sophie L.; Gallier, Suzy; Evison, Felicity; Dillon, Amy; Glampson, Ben; Davies, Quinta; Knight, Joanne; Davis, Cai; George, Michael; Gutteridge, Charles; Sapey, Elizabeth; Denholm, Rachel; Mayer, Erik; Chandrabalan, Vishnu; Stammers, Matt; Mason, Suzanne M; HDRUK UK Regional Linked Data Consortium, -

---

VERSION 1 - REVIEW

**Reviewer**	**1**
**Name**	**Sharwood, Lisa**
**Affiliation**	**University of New South Wales, School of Population Health**
**Date**	**12-Jan-2026**
**COI**	**None**

Thank you for the opportunity to provide peer review for this large body of research aiming to evaluate various factors influencing ED presentations and admissions to hospitals in the NHS, UK. The study asks important questions at a time when many factors require consideration as our health systems are increasingly overwhelmed. Several points require address before the paper can be published; noted under each specific section and overall:

ABSTRACT/ADDITIONAL POINTS

The aim of the study is stated as examining geographic variation in ED attendances and admissions for AEC conditions... yet the methods do not suggest exploration by geospatial unit? This is unclear - please articulate for the reader. Of note, the only further mention in the abstract relates to 'deprived areas' yet we are naive at this point as to how the country was split for this analysis. The 'Methods' uses the word hospital singular to describe '21

acute'... should be plural. Were the data evaluated by hospital, or by local health district or by 'rural/metro' ?? The Discussion of the Abstract neither clarifies this - stating 'variation between hospitals' (does this mean you also looked at hospital bed management practices?) - ... 'including service configuration and primary care access' - does this mean you also looked at GP numbers / care / access?

The Conclusion is somewhat vague and gives only the too oft used line 'more research is needed'.... this may be true but you must be more specific.

Under 'what this study adds' - and 'how this study' - you refer to 'Same Day Emergency Care' - yet the Abstract describes evaluation of AEC. Is this one and the same term? Your conclusions here propose examination of alternative care pathways such as enhanced pre-hospital triage... Does this mean you considered HOW triage was conducted? What about ambulance care? Was this evaluated?

INTRODUCTION

Please clarify if your statistics comparing ED presentation numbers are population adjusted. A quick search shows that over this same period, the population of England increased by roughly 10–11% (from about 53 million in 2011 to ~58–59 million by 2023–24 based on ONS estimates), meaning that a substantial proportion of the reported 23% rise in ED attendances could reflect population growth rather than an increase in per-capita demand... therefore you should clarify whether the figures are population adjusted.... and reference your source of this.

You reference 'hospital bed occupancy' - but should therefore include any relevant evidence as to whether length of stay - as but ONE facet of the NHS management - has been evaluated as part of this project? Overall the Introduction reads as an attempt to layer together multiple system-level problems which do include rising ED attendances, high bed occupancy, ambulance handover delays, declining public satisfaction, inpatient harm, discharge complexity, prevention policy, and short-stay admissions - but then you finally arrive at AEC as the focus of the study. These points are all valid however the Introduction could be tightened to focus on the actual topic at hand and what the study looked at. The discussion of bed occupancy, ambulance delays, and ED crowding is presented as causal background, but your study does not actually evaluate bed capacity, flow interventions, or LOS management strategies, nor does it analyse temporal change in occupancy or ambulance performance. Also, your references to avoidable harm, infections, and functional decline are not clearly linked to the study’s analytic question, which is geographic variation in attendances and admissions for AEC conditions, rather than patient outcomes. I suggest you narrow the scope of your introduction to state the pressure on urgent care, overview briefly the uncertainty around the extent of ED/admission activity relate to AEC conditions and then how this might vary b/w hospitals. By trying to include a whole of system failure narrative - such as bed flows, d/c delays etc - it becomes unclear what the specific gap in the evidence is that YOUR study aims to address.

METHODS

Study Design- succinctly described.

Data Collection: while you have stated your federated approach - and addressed limiations of this later in the manuscript, I believe this explanation relies heavily on implicit UK/NHS data-infrastructure knowledge (e.g. Secure Data Environments, Caldicott Guardians, CDS datasets) and does not explicitly explain why a federated design was necessary or what it means analytically for readers unfamiliar with these systems. I suggest adding one clarifying sentence in the Methods explicitly defining the federated approach and its analytic consequences, particularly given that this is not a universally standard term outside UK/Europe. This will aid the naiive reader from elsewhere :).

You move on to describe 'inclusion criteria' - and include a brief description of the type 1 EDs here - this is a bit confusing - I suggest having a separate section that describes for the naiive reader - the way that the system is set up - what the ED levels are, how many, access etc. Also please clarify whether you included 'did not wait' presentations - as these are still a valid impact on patient management.

The inclusion criteria state that ED records were included for adults with “an unplanned emergency care attendance for a new clinical condition or deterioration of a chronic condition” however no definition is provided of how “new” was operationalised. You have not described any look-back period, nor andy description of linkage to prior attendances, and no explanation of how repeat attendances or ongoing episodes were handled. As written, it is unclear whether “new” reflects a true incident episode based on prior history, the first attendance within the study window, or simply the clinician recorded primary diagnosis at presentation.

This is important to clarify for all readers because the absence (or presence) of a look-back period affects interpretation of attendances as incident demand rather than repeat or unresolved episodes, particularly for chronic and relapsing AEC conditions.

RE: Analysis section: your described analysis appears to be conducted at hospital level, with outcomes expressed as proportions of total ED attendances or total emergency admissions at each site, however this is not stated explicitly and the units of analysis and denominators therefore require clearer description. In addition, no population-based adjustment is undertaken, meaning that variation is assessed relative to hospital activity volumes rather than local catchment population size or composition, and it would be helpful for the authors to clarify this explicitly and acknowledge the implications for interpreting inter-hospital differences... in particular as you are attempting to describe whole of system evaluation here.

Identifiying AEC conditions - please reference the statement re 'these conditions are most commonly managed in an ambulatory care, clinical decision unit or same day emergency

care environment'. Please also clarify whether ALL EDs for which data was collected, use exclusively SNOMED-CT codes - or any use another form of classification.

RESULTS

How do you know that the numbers you record (1,513,480) - are "first-time" ED attendances - if you do not have a look back period? Is this just that these cases did not REpresent in the study period? A look-back period or linkage to prior attendances is described that would allow true identification of first-ever or incident presentations. As it is currently written, this appears to mean first attendances within the study period, rather than first-ever presentations, and I request the authors please clarify this explicitly to avoid misinterpretation, particularly given the implications for repeat attendance and demand analysis.

Further in results - substantial inter-hospital variation is reported across all outcomes, including the proportion of ED attendances and emergency admissions for AEC conditions, admission rates, and short length-of-stay admissions; however, the section does not clearly state that these findings are purely descriptive and unadjusted for differences in case mix, acuity, population served, or coding practices. The large contribution of “condition unspecified” diagnoses to AEC activity is also notable, but the Results do not provide sufficient contextualisation to aid the reader in understanding how diagnostic uncertainty or local coding variation may influence the reported distributions. Once expanded somewhat accordingly, you may pick up some additional comments on this in the Discussion.

DISCUSSION

Reasonable, however some statements imply avoidability or missed opportunities for prevention that go beyond what can be directly supported by the descriptive analyses presented. The Discussion would be strengthened therefore by more clearly distinguishing between conditions that are theoretically amenable to AEC/SDEC and admissions that are demonstrably avoidable, and by aligning conclusions more closely with the study’s observational design and analytic limitations. Please avoid the use of throwaway or generic closing statements like 'more research is needed'....these kinds of statements add little unless they are tied directly to the specific uncertainties revealed by the findings. Given that the study clearly identifies where variation exists (by hospital, age, deprivation, diagnosis, and short length of stay), it is appropriate to expect the Discussion and Conclusions to be more precise about what questions remain unanswered and why - you are able with these findings, once tightened as directed, to propose targeted evidence led research questions that would be more helpful to your (and others) future research.

An important topic which is laudable in its efforts and a timely and valuable multi-site analysis using novel federated methods and high-quality routine data. With clearer methodological clarification, tighter framing of the Introduction, and more disciplined alignment between results and interpretation, the paper has the potential to make a

meaningful contribution to understanding variation in ambulatory emergency care across England.

Happy to review a rework of this paper; thank you for the opportunity to provide this review.

**Reviewer**	**2**
**Name**	**Huang, Xiongfeng**
**Affiliation**	**Fuzhou Medical College, Nanchang University**
**Date**	**14-Jan-2026**
**COI**	**None**

This paper holds significant practical significance and policy reference value, but there is still room for improvement. Specific comments are as follows:

1. The paper mentions "23 hospital sites" while the Methods section states "21 acute hospitals" — consistency between these two should be verified.

2. "the proportion of attendances resulting in admission was similar for females (31.4%; 76,305/242,755) and males (34.2%; 146,290/447,670)"

146,290 ÷ 447,670 ≈ 32.7%, which is inconsistent with the marked 34.2%. There is a data calculation error; please carefully verify all data throughout the paper.

3. Redundancy in the Limitations section:

The original text uses "Finally" twice as an introductory adverb, and the core meaning of the first two sentences is completely repetitive, resulting in redundant expression. Additionally, "it" refers to the plural nouns "diagnosis codes" and "AEC classification" and should be revised to "they".

4. The Data Collection section mentions that "data were extracted, harmonised and analysed at seven regional centres", but fails to specify whether the specific distribution of these seven regional centres covers different geographical regions of England or clarify the specific methods for data "harmonisation". The lack of methodological details affects the reproducibility of the study and the persuasiveness of the sample's representativeness.

5. Insufficient depth in discussion and superficial interpretation of results:

For key findings such as "patients from deprived areas have lower AEC-related attendance and admission rates" and "significant variations in AEC-related indicators among different hospitals", only the phenomena are described without in-depth analysis of the underlying causes.

The Discussion section only references one systematic review, fails to compare the results of this study with similar studies in other countries or regions, and does not explain the

similarities, differences, and reasons between the results of this study and existing research. It is recommended to supplement comparisons with relevant studies.

**Reviewer**	**3**
**Name**	**Bellou, Abdelouahab**
**Affiliation**	**Guangdong Academy of Medical Sciences, Emergency Medicine**
**Date**	**17-Jan-2026**
**COI**	**None**

The authors report an interesting study focused ambulatory emergency care (AEC) with the aim to analyse the geographic variation of ED attendances and emergency admissions for AEC conditions.

The study addresses pressure on the healthcare system in England related to the potentially avoidable emergency care. The study quantifies the significant share of ED attendances and admissions that are for AEC conditions and demonstrates substantial inter-hospital variation. The federated data approach is a notable strength for multi-center research. However, the paper has several limitations reported by the authors which weaken the conclusions and potential impact on the emergency care system.

The positive aspects of the research are:

1. The study addresses key UK NHS operational and strategic challenge (rising demand, avoidable admissions).

2. Analysis of over 1.5 million attendances and 660,000 admissions across 21 hospitals provides a strong descriptive overview. The federated analysis model is innovative and addresses important data governance concerns.

3. Effectively demonstrates significant geographic and demographic variation in AEC-related care, which is a crucial finding for system planners.

4. The methods for identifying AEC conditions and the limitations are generally well-described.

My concerns are:

1. Conceptual & Terminological Ambiguity

• "Potentially Avoidable" vs. "Actually Avoidable": The paper frequently conflates "AEC condition" (a clinical category) with "avoidable attendance/admission." The core limitation is that a diagnostic code alone cannot determine avoidability. A patient with "low-risk chest pain" may require admission due to social conditions, frailty, comorbidities, or lack of clear

diagnostic at presentation in the ED. The discussion acknowledges this, but the abstract and results often use avoidability.

• "Condition Unspecified" as a Major Category: The finding that 25% of AEC attendances were coded as "condition unspecified" is a problem. If this includes "ruled-out" diagnoses (e.g., myocardial infarction excluded), it may represent appropriate ED use for rapid assessment, not necessarily avoidable care which significantly impact negatively the interpretation of the 29.6% number.

2. Some methods limitations

• There is a lack of risk Adjustment: The federated approach precluded adjustment for case-mix. Variation could be driven by differences in population age, frailty, morbidity, and social deprivation rather than service configuration. The deprivation findings (lower AEC rates in more deprived areas) are intriguing but needs adjustment to be interpretable.

• Differences in local coding practices and clinical documentation are reported by the authors but fundamentally limit the comparability between sites. This is a major problem for the validity of the variation observed.

• 21 hospitals included is a good number, but I do not understand how they were selected then the generalizability to all country could be limited.

3. Analysis and results presentation

• Presenting only proportions (e.g., 29.6% of attendances) without contextualizing with crude rates (per 1000 population) limits the understanding of the absolute burden on each hospital.

• The finding that AEC attendances have a higher admission rate (32.7%) than non-AEC attendances (23.2%) is critical but should be discussed more. This directly challenges the assumption that AEC conditions are inherently "less severe" or more avoidable. It suggests that these conditions, have a high acuity and need that often admission.

• Comparing LOS ≤2 days between AEC and non-AEC admissions is less informative than comparing the full distribution of LOS. Many non-AEC admissions (e.g., straightforward surgeries) are planned to be short-stay.

4. Discussion and Conclusions

• The discussion appropriately suggests local factors may influence variation but often leans towards implying they do. The ecological design cannot support causal claims about the effectiveness of community services or SDEC.

• The statement that there is "limited and relatively weak evidence" that AEC/SDEC reduces admissions is correct, but the paper's data do not strengthen this evidence; they merely describe the size of the potential target population.

• The call for further research into drivers of variation and alternative pathways is appropriate but could be more specific (e.g., recommending mixed-methods case studies of high- and low-performing sites).

Suggestions to improve the paper

1. Emphasize that the study identifies a large pool of patients with conditions theoretically suitable for ambulatory pathways, but that actual avoidability requires clinical assessment. Use more prudent language (e.g., "potentially amenable to" instead of "avoidable").

2. I recommend to:

o Provide crude rates alongside proportions.

o Conduct a sensitivity analysis excluding the "condition unspecified" category.

o Analyze the correlation between hospital-level AEC admission rates and short-stay admission rates.

3. Make the Discussion more relevant:

o Discuss the paradoxical finding of higher admission rates for AEC conditions in more depth. What does it mean?

o Clearly separate the description of variation from the interpretation of its causes. Frame the drivers (service configuration, primary care access) as hypotheses for future research.

o Recommend specific study designs for the "future work" section (e.g., detailed patient-level cohort studies with comorbidity adjustment, qualitative work on clinician decision-making).

4. Ensure the abstract reflects the study's descriptive nature and does not overstate the avoidability implied by the diagnostic codes.

This paper provides a valuable descriptive mapping of the emergency care landscape for AEC conditions in England. Its primary contribution is highlighting the scale of the phenomenon and the striking variation between hospitals, which warrants more investigation. The main concern is that readers may misinterpret the diagnostic-code-based "AEC condition" as a proxy for "inappropriate or preventable hospital use," which the study design cannot establish. With more nuanced language and a clearer focus on its descriptive strengths, this paper can be a solid foundation for starting policy debates and guiding more definitive research.

**Reviewer**	**4**
**Name**	**Minotti, Bruno**
**Affiliation**	**University Hospital Basel, Emergency Department**
**Date**	**26-Jan-2026**

COI **None**

Thank you to the authors for submitting this original article to BMJ Open. As the authors describe, demand on emergency care has increased worldwide in recent years, leading to a growing burden of attendances, overcrowding in emergency departments, and overall consequent dissatisfaction for both patients and providers. Accordingly, analyzing possible alternatives to ED care and hospital admission is highly important, which makes this article potentially interesting. The article is overall well written and demonstrates a meticulous design. However, two major concerns would benefit from further consideration.

1. AIM

As the authors clearly state after the abstract, this study emphasizes the problem of unnecessary hospital admissions (“what we know”). They report that “a substantial proportion of ED attendances and acute hospital admissions are for conditions potentially amenable to Same Day Emergency Care or community-based management” (“what this study adds”), which could “advocate for further examination of alternative care pathways, including Same Day Emergency Care, ambulatory care units, virtual wards, and enhanced pre-hospital triage” (“impact of the study”).

However, already in the title, the authors place the primary focus on variation in practice between hospitals rather than on the proportion of potentially avoidable attendances. Similarly, in the abstract, the stated aim of the study is to assess variability, whereas the results predominantly report the proportion of potentially avoidable attendances. In contrast, variation between hospitals is only marginally described. Finally, even in the conclusion, the emphasis returns to the issue of avoidable hospital utilization, leaving the question of variation largely unaddressed.

Finally, although the authors acknowledge inter-site variation in admission criteria, no data are provided to clarify what these criteria are at each hospital. As a result, analysis of the underlying decision-making processes is difficult to extrapolate.

2. Definition of AEC

The authors provide a list of diagnoses for which ambulatory emergency care might be appropriate (Supplementary material). Although this list is derived from a defined concept and supported by a reference, it appears that many of these diagnoses would first require clinical triage, as some cases may clearly necessitate acute ED care and/or hospital admission (e.g. severe cardiac failure, pulmonary embolism with haemodynamic compromise, pneumothorax with severe symptoms, etc.). Therefore, relying solely on diagnostic labels to determine the feasibility of ambulatory emergency care represents a major limitation and may not accurately reflect the cohort of patients who could realistically be managed within an ambulatory emergency care pathway. Although the authors partially acknowledge this issue in the limitations, it appears to represent more than a matter of “clinical nuance or patient preference”.

I fully agree that “identifying high-performing systems with lower avoidable admission rates may yield valuable insights into service models and resource configurations that mitigate unnecessary admissions”. However, the present study does not appear to achieve this aim and offers limited insight into how such systems could be identified. At a minimum, a matched analysis based on the diagnoses selected for ambulatory emergency care could have provided some indication of which patients might realistically be managed within ambulatory emergency care pathways. This approach might also have helped to refine the broadly defined list currently provided by NHS guidance, moving towards a more specific and clinically applicable set of conditions.

MINORS

The authors refer at different points to 21 hospitals and at other points to 23 hospitals. Please clarify the exact number of hospitals included in the study.

VERSION 1 - AUTHOR RESPONSE

Reviewer 1

<u>ABSTRACT/ADDITIONAL POINTS</u>

The aim of the study is stated as examining geographic variation in ED attendances and admissions for AEC conditions... yet the methods do not suggest exploration by geospatial unit? This is unclear - please articulate for the reader. Of note, the only further mention in the abstract relates to 'deprived areas' yet we are naive at this point as to how the country was split for this analysis.

The 'Methods' uses the word hospital singular to describe '21 acute'... should be plural.

Were the data evaluated by hospital, or by local health district or by 'rural/metro' ?? The Discussion of the Abstract neither clarifies this - stating 'variation between hospitals' (does this mean you also looked at hospital bed management practices?) - ... 'including service configuration and primary care access' - does this mean you also looked at GP numbers / care / access?

The Conclusion is somewhat vague and gives only the too oft used line 'more research is needed'.... this may be true but you must be more specific.

Under 'what this study adds' - and 'how this study' - you refer to 'Same Day Emergency Care' - yet the Abstract describes evaluation of AEC. Is this one and the same term? Your conclusions here propose examination of alternative care pathways such as enhanced pre-hospital triage... Does this mean you considered HOW triage was conducted? What about ambulance care? Was this evaluated?

We value this feedback and have amended the abstract to reflect these comments.

INTRODUCTION

Please clarify if your statistics comparing ED presentation numbers are population adjusted. A quick search shows that over this same period, the population of England increased by roughly 10–11% (from about 53 million in 2011 to ~58–59 million by 2023–24 based on ONS estimates), meaning that a substantial proportion of the reported 23% rise in ED attendances could reflect population growth rather than an increase in per-capita demand... therefore you should clarify whether the figures are population adjusted.... and reference your source of this.

We have adjusted the text to reflect that the increase has been observed even after accounting for population growth

You reference 'hospital bed occupancy' - but should therefore include any relevant evidence as to whether length of stay - as but ONE facet of the NHS management - has been evaluated as part of this project? Overall the Introduction reads as an attempt to layer together multiple system-level problems which do include rising ED attendances, high bed occupancy, ambulance handover delays, declining public satisfaction, inpatient harm, discharge complexity, prevention policy, and short-stay admissions - but then you finally arrive at AEC as the focus of the study. These points are all valid however the Introduction could be tightened to focus on the actual topic at hand and what the study looked at. The discussion of bed occupancy, ambulance delays, and ED crowding is presented as causal background, but your study does not actually evaluate bed capacity, flow interventions, or LOS management strategies, nor does it analyse temporal change in occupancy or ambulance performance. Also, your references to avoidable harm, infections, and functional decline are not clearly linked to the study's analytic question, which is geographic variation in attendances and admissions for AEC conditions, rather than patient outcomes. I suggest you narrow the scope of your introduction to state the pressure on urgent care, overview briefly the uncertainty around the extent of ED/admission activity relate to AEC conditions and then how this might vary b/w hospitals. By trying to include a whole of system failure narrative - such as bed flows, d/c delays etc - it becomes unclear what the specific gap in the evidence is that YOUR study aims to address.

We value this feedback and have amended the introduction section to reflect these comments.

METHODS

Data Collection: while you have stated your federated approach - and addressed limiations of this later in the manuscript, I believe this explanation relies heavily on implicit UK/NHS data-infrastructure knowledge (e.g. Secure Data Environments, Caldicott Guardians, CDS datasets) and does not explicitly explain why a federated design was necessary or what it means analytically for readers unfamiliar with these systems. I suggest adding one clarifying sentence in the Methods explicitly defining the federated approach and its analytic consequences, particularly given that this is not a universally standard term outside UK/Europe. This will aid the naiive reader from elsewhere :).

We have added some more detail about the federated approach to the data collection section of the manuscript.

You move on to describe 'inclusion criteria' - and include a brief description of the type 1 EDs here - this is a bit confusing - I suggest having a separate section that describes for the naiive reader - the way that the system is set up - what the ED levels are, how many, access etc. Also please clarify whether you included 'did not wait' presentations - as these are still a valid impact on patient management.

We have changed the terminology from Type 1 ED to major ED and added a sentence saying that mono-specialty emergency care services, minor injury units and walk-in centres were excluded.

We did not exclude 'did not wait' presentations but we also did not look at their impact on the results. We have added a sentence to the inclusion/exclusion criteria to clarify this.

The inclusion criteria state that ED records were included for adults with "an unplanned emergency care attendance for a new clinical condition or deterioration of a chronic condition" however no definition is provided of how "new" was operationalised. You have not described any look-back period, nor any description of linkage to prior attendances, and no explanation of how repeat attendances or ongoing episodes were handled. As written, it is unclear whether "new" reflects a true incident episode based on prior history, the first attendance within the study window, or simply the clinician recorded primary diagnosis at presentation. This is important to clarify for all readers because the absence (or presence) of a look-back period affects interpretation of attendances as incident demand rather than repeat or unresolved episodes, particularly for chronic and relapsing AEC conditions.

Although we did not apply our own look back period there is a field in the routinely collected data that records type of attendance as first unplanned, planned follow-up within 7 days and unplanned follow-up within 7 days. We have clarified this in the Inclusion and Exclusion Criteria. "ED records were excluded if there were for a planned

follow-up or an unplanned follow-up for the same or related clinical condition within 7 days of the first emergency care attendance".

RE: Analysis section: your described analysis appears to be conducted at hospital level, with outcomes expressed as proportions of total ED attendances or total emergency admissions at each site, however this is not stated explicitly and the units of analysis and denominators therefore require clearer description. In addition, no population-based adjustment is undertaken, meaning that variation is assessed relative to hospital activity volumes rather than local catchment population size or composition, and it would be helpful for the authors to clarify this explicitly and acknowledge the implications for interpreting inter-hospital differences... in particular as you are attempting to describe whole of system evaluation here.

We have updated the analysis description to make it clear that the primary unit of analysis is the hospital level and that the denominators for the primary outcomes are the total number of attendances or emergency admissions rather than a population denominator.

We have also updated the limitations section to discuss the lack of case-mix adjustment.

Identifiying AEC conditions - please reference the statement re 'these conditions are most commonly managed in an ambulatory care, clinical decision unit or same day emergency care environment'. Please also clarify whether ALL EDs for which data was collected, use exclusively SNOMED-CT codes - or any use another form of classification.

We have modified the statement concerning the conditions and added two references.

RESULTS

How do you know that the numbers you record (1,513,480) - are "first-time" ED attendances - if you do not have a look back period? Is this just that these cases did not represent in the study period? A look-back period or linkage to prior attendances is described that would allow true identification of first-ever or incident presentations. As it is currently written, this appears to mean first attendances within the study period, rather than first-ever presentations, and I request the authors please clarify this explicitly to avoid misinterpretation, particularly given the implications for repeat attendance and demand analysis.

Although we did not apply our own look back period there is a field in the routinely collected data that records type of attendance as first unplanned, planned follow-up within 7 days and unplanned follow-up within 7 days. We have clarified this in the Inclusion and Exclusion Criteria. "ED records were excluded if there were for a planned

follow-up or an unplanned follow-up for the same or related clinical condition within 7 days of the first emergency care attendance”.

Further in results - substantial inter-hospital variation is reported across all outcomes, including the proportion of ED attendances and emergency admissions for AEC conditions, admission rates, and short length-of-stay admissions; however, the section does not clearly state that these findings are purely descriptive and unadjusted for differences in case mix, acuity, population served, or coding practices. The large contribution of “condition unspecified” diagnoses to AEC activity is also notable, but the Results do not provide sufficient contextualisation to aid the reader in understanding how diagnostic uncertainty or local coding variation may influence the reported distributions. Once expanded somewhat accordingly, you may pick up some additional comments on this in the Discussion.

We have updated the methods section to make it clear that all analyses were descriptive and no population-based adjustments were performed. We have also included this in our updated limitations section.

DISCUSSION

Reasonable, however some statements imply avoidability or missed opportunities for prevention that go beyond what can be directly supported by the descriptive analyses presented. The Discussion would be strengthened therefore by more clearly distinguishing between conditions that are theoretically amenable to AEC/SDEC and admissions that are demonstrably avoidable, and by aligning conclusions more closely with the study’s observational design and analytic limitations. Please avoid the use of throwaway or generic closing statements like 'more research is needed'....these kinds of statements add little unless they are tied directly to the specific uncertainties revealed by the findings. Given that the study clearly identifies where variation exists (by hospital, age, deprivation, diagnosis, and short length of stay), it is appropriate to expect the Discussion and Conclusions to be more precise about what questions remain unanswered and why - you are able with these findings, once tightened as directed, to propose targeted evidence led research questions that would be more helpful to your (and others) future research.

This is helpful feedback and we have revised the discussion section accordingly.

Reviewer 2

1. The paper mentions "23 hospital sites" while the Methods section states "21 acute hospitals" — consistency between these two should be verified.

Apologies for this inconsistency. There are 21 hospitals included in the analysis and the manuscript has been updated to reflect this.

2. "the proportion of attendances resulting in admission was similar for females (31.4%; 76,305/242,755) and males (34.2%; 146,290/447,670)"

146,290 ÷ 447,670 ≈ 32.7%, which is inconsistent with the marked 34.2%. There is a data calculation error; please carefully verify all data throughout the paper.

The numbers reported were for all attendances, this has now been corrected to show proportion for males: 34.2%; 69,990/204,860

3. Redundancy in the Limitations section:

The original text uses "Finally" twice as an introductory adverb, and the core meaning of the first two sentences is completely repetitive, resulting in redundant expression. Additionally, "it" refers to the plural nouns "diagnosis codes" and "AEC classification" and should be revised to "they".

We have amended the text.

4. The Data Collection section mentions that "data were extracted, harmonised and analysed at seven regional centres", but fails to specify whether the specific distribution of these seven regional centres covers different geographical regions of England or clarify the specific methods for data "harmonisation". The lack of methodological details affects the reproducibility of the study and the persuasiveness of the sample's representativeness.

We have added an extra column to Table 1 indicating the region in which each analysis centre is based and added a sentence to the data collection section describing that the centres cover a wide geography incorporating urban, semi-urban and rural locations.

We have added detail to the methods section about the data processing.

5. Insufficient depth in discussion and superficial interpretation of results:

For key findings such as "patients from deprived areas have lower AEC-related attendance and admission rates" and "significant variations in AEC-related indicators among different hospitals", only the phenomena are described without in-depth analysis of the underlying causes.

Given study design and the nature of the data collected we are unable to do in-depth analysis of the underlying causes. We have added a comment to the limitations section of the paper highlighting that we were unable to do this.

The Discussion section only references one systematic review, fails to compare the results of this study with similar studies in other countries or regions, and does not explain the

similarities, differences, and reasons between the results of this study and existing research. It is recommended to supplement comparisons with relevant studies.
We have added reference to other relevant studies and expanded the reference list accordingly. This section highlights where our study has synergies with previous studies.

Reviewer 3

1. Conceptual & Terminological Ambiguity

• "Potentially Avoidable" vs. "Actually Avoidable": The paper frequently conflates "AEC condition" (a clinical category) with "avoidable attendance/admission." The core limitation is that a diagnostic code alone cannot determine avoidability. A patient with "low-risk chest pain" may require admission due to social conditions, frailty, comorbidities, or lack of clear diagnostic at presentation in the ED. The discussion acknowledges this, but the abstract and results often use avoidability.

We have tried to reduce confusion in relation to the terminology we use in the paper. We have also made it clear that there are some potentially avoidable admissions that are clinically necessary and therefore estimates of avoidable admissions should be treated cautiously.

• "Condition Unspecified" as a Major Category: The finding that 25% of AEC attendances were coded as "condition unspecified" is a problem. If this includes "ruled-out" diagnoses (e.g., myocardial infarction excluded), it may represent appropriate ED use for rapid assessment, not necessarily avoidable care which significantly impact negatively the interpretation of the 29.6% number.
We strongly believe that presenting this category is important as it reflects potential diagnostic uncertainty which has previously been identified as a driving factor for how potentially avoidable admissions are managed. It also reflects the challenges with using medical coding systems in relation to undifferentiated urgent care patients, where a diagnosis can often be made sometime in the future, following their emergency department attendance.

2. Some methods limitations

• There is a lack of risk Adjustment: The federated approach precluded adjustment for case-mix. Variation could be driven by differences in population age, frailty, morbidity, and social deprivation rather than service configuration. The deprivation findings (lower AEC rates in more deprived areas) are intriguing but needs adjustment to be interpretable.

We have updated the limitations section to discuss the lack of case-mix adjustment.

• Differences in local coding practices and clinical documentation are reported by the authors but fundamentally limit the comparability between sites. This is a major problem for the validity of the variation observed.
This is a well known challenge in analysing real world data and we agree potentially impacts on the study findings. We have updated our limitations section to reflect this.

• 21 hospitals included is a good number, but I do not understand how they were selected then the generalizability to all country could be limited.

We have added a sentence referring to the geographical spread of the hospitals included in the study. The included hospitals were a convenience sample driven by the availability of data for analysis.

3. Analysis and results presentation

• Presenting only proportions (e.g., 29.6% of attendances) without contextualizing with crude rates (per 1000 population) limits the understanding of the absolute burden on each hospital.

Hospitals in England do not have well defined catchment areas and therefore it is difficult to define the population that they serve. As we do not have a denominator, we are unable to calculate crude rate per 1000 population, and this is why we analysed the proportion of attendances.

• The finding that AEC attendances have a higher admission rate (32.7%) than non-AEC attendances (23.2%) is critical but should be discussed more. This directly challenges the assumption that AEC conditions are inherently "less severe" or more avoidable. It suggests that these conditions, have a high acuity and need that often admission.

We have added some text in the discussion section relating to this paradoxical finding, and we have suggested some of the reasons for these findings.

• Comparing LOS ≤2 days between AEC and non-AEC admissions is less informative than comparing the full distribution of LOS. Many non-AEC admissions (e.g., straightforward surgeries) are planned to be short-stay.

In the UK, non-AEC admission often do not have any planned LOS in hospital, although often the intention is to aim for LOS as short as possible in all acute admissions. We took a pragmatic decision to evaluate LOS as a cut off rather than a continuum in order to make a clear distinction between those who stay less than 3 days and might be considered potentially avoidable when compared with those staying longer.

4. Discussion and Conclusions

• The discussion appropriately suggests local factors may influence variation but often leans towards implying they do. The ecological design cannot support causal claims about the effectiveness of community services or SDEC.

We have updated our discussion section.

• The statement that there is "limited and relatively weak evidence" that AEC/SDEC reduces admissions is correct, but the paper's data do not strengthen this evidence; they merely describe the size of the potential target population.

We have updated our discussion section.

• The call for further research into drivers of variation and alternative pathways is appropriate but could be more specific (e.g., recommending mixed-methods case studies of high- and low-performing sites).

We have updated our conclusions section to say that future work should explore the variation observed between hospitals.

Suggestions to improve the paper

1. Emphasize that the study identifies a large pool of patients with conditions theoretically suitable for ambulatory pathways, but that actual avoidability requires clinical assessment. Use more prudent language (e.g., "potentially amenable to" instead of "avoidable").

We have done this in the introduction and discussion sections

2. I recommend to:
o Provide crude rates alongside proportions.

Hospitals in England do not have well defined catchment areas and therefore it is difficult to define the population that they serve. As we do not have a denominator we are unable to calculate crude rate per 1000 population and this is why we analysed the proportion of attendances.

o Conduct a sensitivity analysis excluding the "condition unspecified" category.

Unfortunately due to the way in which the data were collected we are unable to exclude “condition unspecified” to conduct a sensitivity analysis.

o Analyze the correlation between hospital-level AEC admission rates and short-stay admission rates.

The Spearman rank correlation between the proportion of attendances for AEC that result in admission and proportion of acute admissions for AEC with length of stay less than two days is -0.05 (95% CI: -0.47 to 0.39). This result has been added to the paper.

3. Make the Discussion more relevant:
o Discuss the paradoxical finding of higher admission rates for AEC conditions in more depth. What does it mean?

o Clearly separate the description of variation from the interpretation of its causes. Frame the drivers (service configuration, primary care access) as hypotheses for future research.

o Recommend specific study designs for the "future work" section (e.g., detailed patient-level cohort studies with comorbidity adjustment, qualitative work on clinician decision-making).

Thankyou we have made reference to these bullet points

4. Ensure the abstract reflects the study's descriptive nature and does not overstate the avoidability implied by the diagnostic codes.

We have revised the abstract

Reviewer 4

1. AIM
As the authors clearly state after the abstract, this study emphasizes the problem of unnecessary hospital admissions (“what we know”). They report that “a substantial proportion of ED attendances and acute hospital admissions are for conditions potentially amenable to Same Day Emergency Care or community-based management” (“what this study adds”), which could “advocate for further examination of alternative care pathways, including Same Day Emergency Care, ambulatory care units, virtual wards, and enhanced pre-hospital triage” (“impact of the study”).

However, already in the title, the authors place the primary focus on variation in practice between hospitals rather than on the proportion of potentially avoidable attendances. Similarly, in the abstract, the stated aim of the study is to assess variability, whereas the results predominantly report the proportion of potentially avoidable attendances. In contrast, variation between hospitals is only marginally described. Finally, even in the conclusion, the emphasis returns to the issue of avoidable hospital utilization, leaving the question of variation largely unaddressed.

Finally, although the authors acknowledge inter-site variation in admission criteria, no data are provided to clarify what these criteria are at each hospital. As a result, analysis of the underlying decision-making processes is difficult to extrapolate.

We have revised the text throughout to reflect these comments.

2. Definition of AEC

The authors provide a list of diagnoses for which ambulatory emergency care might be appropriate (Supplementary material). Although this list is derived from a defined concept and supported by a reference, it appears that many of these diagnoses would first require clinical triage, as some cases may clearly necessitate acute ED care and/or hospital admission (e.g. severe cardiac failure, pulmonary embolism with haemodynamic compromise, pneumothorax with severe symptoms, etc.). Therefore, relying solely on diagnostic labels to determine the feasibility of ambulatory emergency care represents a major limitation and may not accurately reflect the cohort of patients who could realistically be managed within an ambulatory emergency care pathway. Although the authors partially acknowledge this issue in the limitations, it appears to represent more than a matter of "clinical nuance or patient preference".

We have expanded on this in the discussion section, especially making the point that AEC conditions represent a heterogeneous group of presentations, many of which may appropriately require inpatient admission depending on patient complexity, diagnostic uncertainty, or local service configuration. Our findings therefore describe the potential population for ambulatory pathways rather than the proportion of admissions that could safely have been avoided.

I fully agree that "identifying high-performing systems with lower avoidable admission rates may yield valuable insights into service models and resource configurations that mitigate unnecessary admissions". However, the present study does not appear to achieve this aim and offers limited insight into how such systems could be identified. At a minimum, a matched analysis based on the diagnoses selected for ambulatory emergency care could have provided some indication of which patients might realistically be managed within ambulatory emergency care pathways. This approach might also have helped to refine the broadly defined list currently provided by NHS guidance, moving towards a more specific and clinically applicable set of conditions.

The study was conducted to present an overall descriptive picture of patients attending the ED with AEC conditions. It sought to describe this population and also understand how varied admission rates are between hospitals. Whilst we agree with the suggestion that a matched analysis would be insightful, it was not part of our original plan for this study.

The authors refer at different points to 21 hospitals and at other points to 23 hospitals. Please clarify the exact number of hospitals included in the study.

Apologies for this inconsistency. There are 21 hospitals included in the analysis and the manuscript has been updated to reflect this.

VERSION 2 - REVIEW

**Reviewer**	**3**
**Name**	**Bellou, Abdelouahab**
**Affiliation**	**Guangdong Academy of Medical Sciences, Emergency Medicine**
**Date**	**07-Apr-2026**
**COI**	

Authors revised appropriately the manuscript.

**Reviewer**	**4**
**Name**	**Minotti, Bruno**
**Affiliation**	**University Hospital Basel, Emergency Department**
**Date**	**16-Apr-2026**
**COI**	

I commend the authors for this thorough review. All comments have been addressed, substantially improving the manuscript.
